# Draft Genome Sequence of the Endophyte *Bacillus mycoides* Strain GM5LP Isolated from *Lolium perenne*

**DOI:** 10.1128/genomeA.01517-17

**Published:** 2018-01-25

**Authors:** Jacqueline Hollensteiner, Anja Poehlein, Sandra Granzow, Heiko Liesegang, Rolf Daniel, Stefan Vidal, Franziska Wemheuer

**Affiliations:** a Genomic and Applied Microbiology & Göttingen Genomics Laboratory, Institute of Microbiology and Genetics, University of Göttingen, Göttingen, Germany; b Agricultural Entomology, Department of Crop Sciences, University of Göttingen, Göttingen, Germany

## Abstract

*Bacillus mycoides* GM5LP is a Gram-positive endophytic bacterium isolated from aerial plant tissues of *Lolium perenne* L. The 6.0-Mb draft genome harbors 6,132 protein-coding sequences, some of which might be involved in the biosynthesis of antimicrobial substances.

## GENOME ANNOUNCEMENT

Species of the *Bacillus* genus are common members of the plant microbiome (1, 2). Several bacilli are important plant growth-promoting bacteria, as they produce a wide range of antimicrobial and antifungal substances and thus can act as biological control agents of various phytopathogens (3–5). The genome of the endophytic *Bacillus mycoides* strain GM5LP was sequenced to explore its genomic features and its potential as a biocontrol agent.

We isolated *B. mycoides* GM5LP from surface-sterilized aerial tissues of healthy *Lolium perenne* plants. Genomic DNA was extracted using the MasterPure complete DNA purification kit (Epicentre, Madison, WI, USA). The obtained DNA was used to generate Illumina shotgun paired-end sequencing libraries. Sequencing was performed employing a MiSeq system and the MiSeq reagent kit version 3 (600 cycles), as recommended by the manufacturer (Illumina, San Diego, CA, USA). Quality filtering was performed using Trimmomatic version 0.32 (6) and resulted in 2,866,928 paired-end reads. *De novo* genome assembly was performed with the SPAdes genome assembler version 3.8.0 (7). The assembly resulted in 101 contigs (>500 bp), with an average coverage of 93×. The assembly was validated and the read coverage determined with Qualimap version 2.1 (8).

The draft genome of *B. mycoides* GM5LP consisted of 6,016,834 bp, with an overall GC content of 35.08%. Gene prediction and annotation were performed using Prokka (Rapid Prokaryotic Genome Annotation) version 1.11 (9). The draft genome harbored 15 rRNA genes, 96 tRNA genes, 2,355 protein-coding genes with functional predictions, and 3,777 genes coding for hypothetical proteins. Multilocus sequence typing (MLST) based on seven housekeeping genes (*glp, gmk, ilvD, pta, pur, pyc,* and *tpi*) was performed according to Priest et al. (10). The analysis revealed that strain GM5LP clusters with *Bacillus weihenstephanensis* within the *Bacillus cereus sensu lato* group. *Bacillus weihenstephanensis* was recently reclassified as a heterotypic synonym of *Bacillus mycoides* (11).

Secondary metabolite gene prediction using antiSMASH 3.0.5 (12) resulted in 47 predicted gene clusters, including bacteriocin, terpene, lantipeptide, lassopeptide, and siderophore gene clusters. Three novel nonribosomal polyketide synthetase (NRPS) clusters were identified. Genes involved in bacteriocin production might be beneficial for plant growth (13) and the control of other bacteria (14). A siderophore gene cluster similar to a catecholate petrobactin cluster known to be involved in virulence of *Bacillus anthracis* was identified (15). A lassopeptide gene cluster with 100% of genes exhibiting similarity to a paeninodin (16, 17) biosynthetic gene cluster known from *Paenibacillus dendritiformis* was detected. Paeninodin belongs to the ribosomally synthesized and posttranslationally modified peptides (RiPPs) that harbor pharmacologically relevant compounds, as they exhibit a wide range of antimicrobial or antiviral activities (18). A complete biosynthesis gene cluster was identified for polyhydroxyalkanoate (PHA), a compound often used in medicine or agriculture (19). Several plant growth-promoting bacteria produce PHAs, which hold advantageous characteristics of enhanced root colonization or plant growth promotion (20). The genome sequence of *B. mycoides* strain GM5LP will facilitate further studies on the potential of this bacterium as a producer of antimicrobial substances.

### Accession number(s).

The whole-genome shotgun project has been deposited at DDBJ/ENA/GenBank under the accession number MKZP00000000. The version described here is version MKZP01000000.
